# The efficacy of PRECEDE-PROCEED model-based interventions on HbA1c and self-management in type 2 diabetes patients: a systematic review and meta-analysis

**DOI:** 10.1186/s12889-025-23073-9

**Published:** 2025-05-29

**Authors:** Yan Tang, Kim Lam Soh, Wan Ying Gan, Junjun Zhou, Kim Geok Soh

**Affiliations:** 1 Department of Nursing, University Putra Malaysia, Selangor, Serdang Malaysia; 2 Faculty of Nursing, Jiangsu Medical College, Yancheng, Jiangsu China; 3 Department of Endocrinology, Yancheng No.1 People’s Hospital, Affiliated Hospital of Medical School, Nanjing University, Nanjing, China; 4 Department of Nutrition, University Putra Malaysia, Selangor, Serdang Malaysia; 5 Department of Sports Studies, University Putra Malaysia, Selangor, Serdang Malaysia

**Keywords:** PRECEDE-PROCEED model, Type 2 diabetes, Self-management, Meta-analysis

## Abstract

**Background:**

Type 2 diabetes is a global public health challenge characterized by high prevalence and poor self-management outcomes. The PRECEDE-PROCEED model is a well-known conceptual widely used to promote health in chronic diseases. This meta-analysis evaluated the efficacy of interventions based on PRECEDE-PROCEED model in reducing Glycated Hemoglobin A1c (HbA1c) and enhancing self-management in patients with type 2 diabetes.

**Methods:**

Nine online databases—PubMed, Cochrane Library, Embase, PsycINFO, Scopus, CINAHL, Web of Science, CNKI, and WANFANG—were searched from inception to June 2024. Randomized controlled trials (RCTs) and quasi-experimental studies (QESs) were identified using keywords related to the PRECEDE-PROCEED model, type 2 diabetes, and self-management. Review Manager 5.4 was used for meta-analysis and the 95% confidence intervals (CIs) was calculated for standardized mean differences (SMDs) or weighted mean differences (WMDs).

**Results:**

Fourteen studies (11 RCTs and 3 QESs) involving 2,478 patients met the eligibility criteria. Interventions significantly reduced HbA1c, with progressive improvements over time: WMD = -0.41 (95% CI: -0.58 to -0.24) at 1 month, -0.50 (95% CI: -0.67 to -0.33) at 3 months, and -0.63 (95% CI: -0.93 to -0.33) at 6 months. Significant improvements were also observed in total self-management scores (SMD = 2.53; 95% CI: 1.14to 3.91) and in key PRECEDE-PROCEED model variables, including knowledge, attitudes, self-efficacy, reinforcing, and enabling factors, though high heterogeneity was noted.

**Conclusions:**

The PRECEDE-PROCEED model is an effective framework for reducing HbA1c and enhancing self-management among patients with type 2 diabetes. Future research should focus on standardizing intervention protocols and developing unified outcome measures to improve reproducibility and cross-study comparability.

**Trial registration:**

The PROSPERO registration ID is CRD42024600814.

## Background

Diabetes presents a growing health and economic burden on individuals and societies globally [1]. In 2021, approximately 537 million individuals were dealing with diabetes, and the prevalence is projected to rise, reaching an estimated 643 million by 2030 and 783 million by 2045 [2]. Type 2 diabetes mellitus (T2DM), which accounts for 90–95% of all diabetes cases, is characterized by hyperglycaemia. This condition can contribute to various microvascular complications, such as retinopathy, neuropathy, and nephropathy. Hyperglycemia is also associated with macrovascular complications, including coronary artery disease and cerebrovascular disease [3]. Moreover, T2DM is a major global cause of mortality, with diabetes-related deaths rising significantly from 1.5 million in 2012 to 6.7 million in 2021 [1].

Self-management is a practical, proactive process in which individuals take responsibility for managing their own health [4]. For people with T2DM, this includes monitoring and controlling key health indicators such as blood glucose levels, cholesterol, blood pressure, and weight, as well as practicing proper foot care, engaging in regular physical activity, and adhering to prescribed diet and medication [5]. Health education is now widely recognized as a critical component of diabetes care, as it encourages patients to actively manage their condition and adopt healthier lifestyle choices. As patient involvement in healthcare increases, effective education becomes essential for enhancing self-management practices. By employing effective teaching methods, healthcare providers can help patients achieve better health outcomes [5].

Theories and models provide a structured framework to guide educational interventions, enhance their effectiveness and tailor them to individual needs [6]. One such model is the PRECEDE-PROCEED model, also known as the Greene model, constructed by health educator Lawrence W. Green. The PRECEDE-PROCEED model, which consists of the Predisposing, Reinforcing, and Enabling Constructs in Educational Diagnosis and Evaluation (PRECEDE) and the Policy, Regulatory, and Organizational Constructs in Educational and Environmental Development (PROCEED), is a widely recognized framework in public health that emphasizes a participatory approach to planning and evaluating health promotion initiatives [7]. It organizes health promotion practice into nine sequential steps and identifies enabling, reinforcing, and predisposing factors that influence behaviour during educational diagnostics [8].

In recent years, scholars worldwide have increasingly investigated the impact of health education driven by the PRECEDE-PROCEED model on metabolism, quality of life, and self-efficacy among individuals with T2DM [9–11]. However, considerable variation exists in the implementation of PRECEDE-PROCEED model-based interventions [12]. Additionally, there is a lack of meta-analyses and comprehensive assessments addressing the impact of these interventions on self-management in patients with T2DM [13]. This meta-analysis aims to evaluate the effectiveness of PRECEDE-PROCEED model-based interventions in T2DM management, focusing on key outcomes such as HbA1c and self-management. The findings seek to fill existing research gaps and provide robust evidence for the model's application in diabetes education.

## Methods

This review, CRD42024600814, has been filed at the International Prospective Register of Systematic Reviews (PROSPERO) and executed in compliance with the Preferred Reporting Items for Systematic Reviews and Meta-Analyses (PRISMA) Protocols [14].

### Search strategy

Nine online databases PubMed, Cochrane Library, Embase, PsycINFO, Web of Science, Scopus, CINAHL, CNKI, and WANFANG were searched using the predefined search terms from inception to June 2, 2024. The search formula was as follows: ("Precede-Proceed"OR"PrecedeProceed"OR"Precede Proceed") AND ("Diabetes Mellitus, Type 2"[MeSH] OR"Type 2 Diabetes"OR"T2DM"OR"NIDDM"OR"Diabetes Mellitus, Noninsulin-Dependent"OR"Maturity-Onset Diabetes Mellitus"OR"MODY"OR"Adult-Onset Diabetes Mellitus") AND ("Self Care"[MeSH] OR"Self Management"[MeSH] OR"self-manage*"OR"self-management"OR"self-regulation"OR"self-monitoring"OR"medication adherence"OR"dietary adherence"OR"weight loss"OR"glycemic control"OR"hemoglobin A1 C"OR"fasting glucose"OR"blood glucose").

### Inclusion and exclusion criteria

This review included quasi-experimental studies (QESs) and randomized controlled trials (RCTs) applying the PRECEDE-PROCEED model to patients with T2DM, published in English or Chinese. QESs were included alongside RCTs to capture a broader range of evidence, particularly in real-world settings where randomization may not be feasible, while RCTs provided high-quality experimental data. Eligible studies were required to report glycemic control or self-management, as the PRECEDE-PROCEED model evaluates both behavioral (self-management) and physiological (glycemic control) outcomes, with the former often being a prerequisite for the latter. The inclusion criteria were: (a) QESs or RCTs; (b) diagnosis of T2DM; (c) PRECEDE-PROCEED model-based interventions; and (d) self-management or glycemic control as outcome measures. The exclusion criteria were: (a) non-original research, such as review articles, editorials, letters, and conference proceedings; (b) studies that did not differentiate between T2DM and type 1 diabetes; and (c) articles lacking full text.2.

### Study selection and screening

Titles and abstracts of all identified studies were screened, and duplicate records were removed. Two researchers independently assessed the full texts for eligibility, and a third researcher resolved any disagreement.

### Data extraction

Data extraction was performed using a custom form developed by three researchers. Extracted data included: (a) surname of the first author; (b) year of the publication and the study location; (c) participant characteristics; (d) intervention details(duration, delivery method, setting, and assessment time points); (e) outcomes; (f) main findings.

### Risk of bias assessment

Two independent researchers assessed the quality of the included studies. The risk of bias in RCTs was evaluated using the Cochrane Risk of Bias tool [15], which categorizes bias into three levels: low, unclear, and high risk. The evaluation considered several factors, including random sequence generation, allocation concealment, blinding of participants and personnel, blinding of outcome assessment, incomplete outcome data, selective reporting, and other potential sources of bias. The risk of bias in QESs was assessed using the Nonrandomized Studies of Interventions (ROBINS-I) tool [16] was applied to assess risk of bias in QESs, which covers the following domains: confounding, participant selection, intervention classification, deviations from intended interventions, missing data, outcome measurement, and selective reporting. Additionally, ROBINS-I allows an optional assessment of the bias direction for each category.

### Data synthesis and analysis

For qualitative synthesis, data were extracted and analyzed on participants, implementation details of the PRECEDE-PROCEED model, outcome measures, and main findings. Meta-analyses were conducted on outcomes, including HbA1c, total self-management scores, and key variables of the PRECEDE-PROCEED model. Data were entered into Review Manager 5.4, a software developed by the Cochrane Collaboration, according to each study's design and outcome metrics. Continuous variables were reported with corresponding *p*-values and 95% confidence intervals (CIs), and effect sizes were calculated using Standardized Mean Difference (SMD) or Mean Difference (MD). A random-effects model was applied to account for statistical heterogeneity, and the *I*^2^ statistic was used to quantify it.

## Results

### Study selection process

**Fig. 1:**
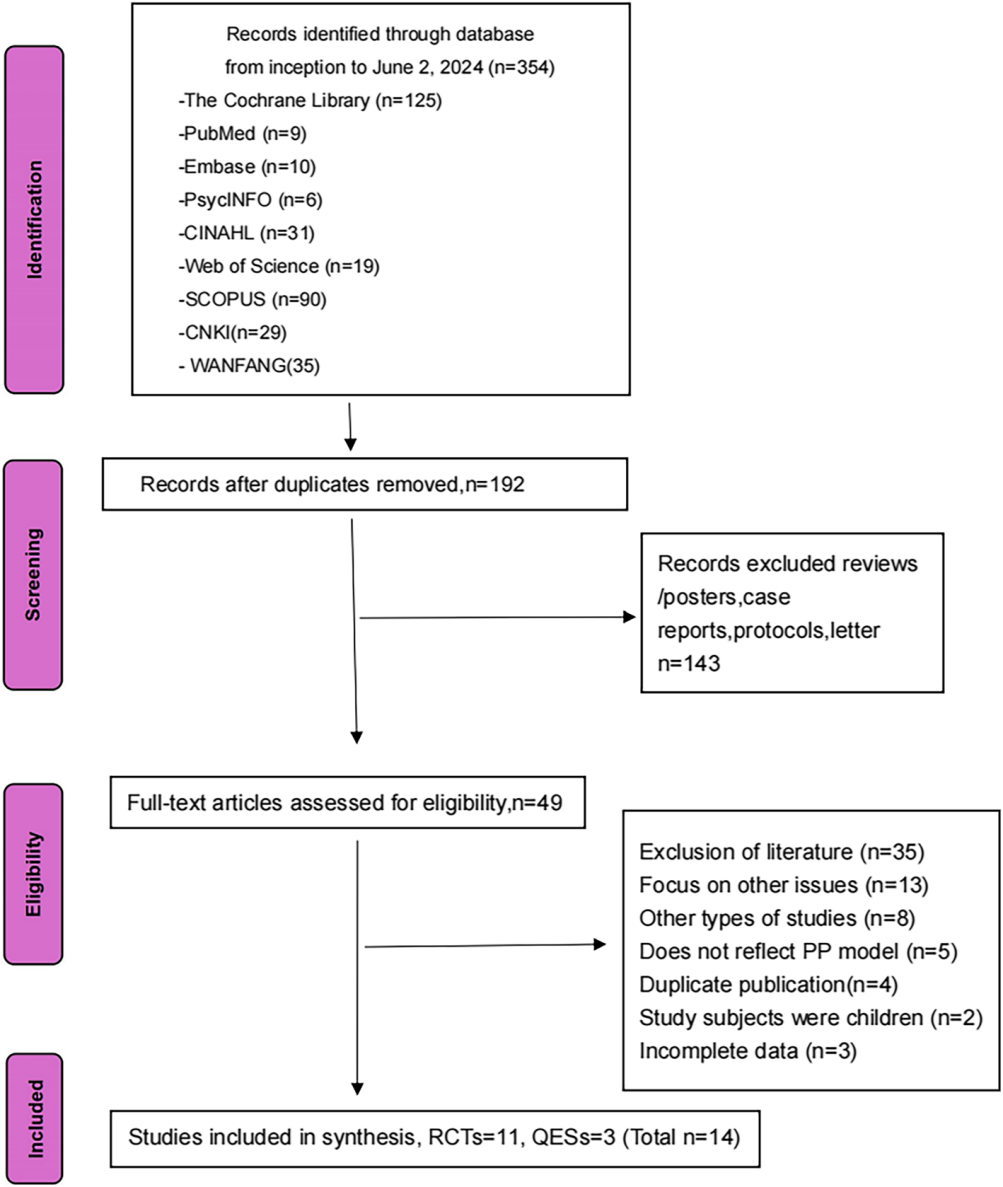
Flow diagram of study selection process

A total of 354 records were retrieved from nine databases. After removing duplicates, 192 records remained for title and abstract screening. Subsequently, 49 papers were selected for full-text evaluation, of which 14 studies met the inclusion criteria. All included studies detailed the intervention methods and clearly defined outcome measures. Each study also reported the baseline data comparability between the intervention and control groups. The study selection process is summarized in the flowchart shown in Fig. 1.

### Characteristics of included studies

Among the fourteen eligible studies, all were published between 2011 and 2023, with eleven (78.6%) being RCTs and three (21.4%) QESs. These studies were conducted in four countries: six in China (42.3%), six in Iran (42.3%), one in Spain (7.1%), and one in the United States (7.1%). The study settings included diabetes clinics (*n* = 5), the endocrinology department (*n* = 4), communities (*n* = 3), and health centers (*n* = 2). A total of 2,478 patients with T2DM were involved, with sample sizes ranging from 66 to 608. Among these studies, one focused on elderly patients over 60 years [17], one on female patients [12], one on Asian Americans [18], and one on empty-nest older adults [19]. The remaining studies included general populations of patients with T2DM. The ages of the patients ranged from 48.7 to 68.1 years, with a mean age of 59.81 years. Table 1 presents the characteristics of the 14 included studies.

**Table 1:** Study characteristics

Author, year, country	Design	Participants			Intervention				Outcomes	Main findings
		**Sample size**		**Age**	**Duration**	**Settings**	**Intervention group delivery way**	**Assessment time points**		
		IG	CG							
Li et al 2023 China [19]	RCT	48	50	67.2 ± 5.24	6 months 60–90 min monthly lectures	Community	Health education lectures, delivery message, in-person meetings, peer support groups, family visits, or follow-up via telephone	3 and 6 months post-intervention	HbA1c, self-management, FPG, 2-h PBG, BMI, BP	PRECEDE-PROCEED model-based education led to significantly greater improvements in self-management and glycemic control in older type 2 diabetes patients living in empty-nest situations
Khani et al 2023 Iran [20]	RCT	150	150	IG: 52.36 CG: 54.11	3 months ten 50–55 min lectures every two weeks	Diabetes clinics	Lectures, question-and-answer sessions, group discussions, practical demonstrations, video presentations, and PowerPoint slides, WhatsApp motivational messages and a brochure	3 months post-intervention	PP model variables, self-management, BP	PRECEDE-PROCEED model-based education led to significant improvements in lifestyle changes, self-management, and hypertension control among diabetes patients
Nejhaddadgar et al 2019 Iran [11]	RCT	43	43	IG: 55.09 ± 13.41 CG: 56.30 ± 10.62	2 months eight weekly sessions	Diabetes clinics	Structured weekly sessions, two-session training workshops for families and health workers	6 months post-intervention	PP model variables, self-management, BMI	PRECEDE-PROCEED model-based education substantially enhances PP variables and self-management behaviors in type 2 diabetes patients
Hosseiniet al 2017 Iran [21]	RCT	53	53	IG: 51.55 ± 8.30 CG: 58.09 ± 1.60	1 month four 120-min weekly sessions	Diabetes clinics	Workshops including lectures, consultations, group discussions, provision of health education booklets and necessary guidance, family support	1, 3, and 6 months post-intervention	HbA1c, PP model variables, BMI	PRECEDE-PROCEED model-based education significantly improves PP variables and self-management behaviors among patients with T2DM
Kimet al 2015 America [18]	RCT	105	104	IG: 59.1 ± 8.4 CG: 58.3 ± 8.5	12 months six 120-min weekly sessions; twelve monthly motivational counseling	Community	Group education involving multimedia presentations, teach-back methods, role-playing, and group discussions, along with self-monitoring; motivational counseling	3, 6, 9 and 12 months post-intervention	HbA1c, self-management, QoL, lipids levels, BP	SHIP-DM effectively improves diabetes control, diabetes-related psychobehavioral outcomes, self-efficacy in diabetes self-management, diabetes knowledge, and diabetes-related quality of life scores among Asian Americans
Salinero-Fort et al 2011 Spain [22]	RCT	300	300	IG: 66.06 CG: 67.28	24 months 0 and 1 visit at month 1; 2 to 9 follow-up visits every 3 months	Health centers	Self-monitoring of blood glucose, guidance on physical exercise, diet, medication adherence, and smoking cessation	After the 24-months follow-up	HbA1c, lipids levels, BP, BMI	PRECEDE health education model significantly improved HbA1c and SBP levels and enhanced compliance with most control criteria, with the exception of LDL cholesterol, in T2DM patients
Mei et al 2019 China [23]	RCT	57	55	IG: 61.5 ± 8.5 CG: 61.2 ± 9.7	6 months telephone and outpatient review follow-up monthly	Endocrinology department	Health records, information brochures, blood glucose and lipid monitoring diaries, medication box reminders, family support and formation of patient groups	1, 3, and 6 months post-intervention	HbA1c, self-management, FPG, 2-h PBG, QoL	PRECEDE-PROCEED model-based health education demonstrates significant benefits in controlling blood glucose, improving self-management behaviors, and improving quality of life in type diabetics
Zhang et al 2020 China [24]	RCT	54	54	IG: 52.32 ± 6.68 CG: 52.67 ± 5.95	6 months follow up	Endocrinology department	Conduct comprehensive patient assessment based on the PP Model, set intervention goals, and provide personalized dietary guidance	6 months post-intervention	HbA1c, self-management, FPG, 2-h PBG	PRECEDE model combined with dietary guidance significantly enhanced health knowledge, self-management skills, blood glucose levels and reduced the incidence of chronic complications in T2DM patients
Li 2015 China [17]	RCT	33	33	IG: 68.18 ± 4.50 CG: 67.33 ± 4.66	3 months four 40 min lectures; four < 30 min face-to-face instruction	Endocrinology department	Based on self-management misconceptions, in-hospital lectures including PPT presentations, case sharing, group discussions and online media; post-discharge face-to-face instruction and 3-day blood glucose profile	Post-intervention and 3 months thereafter	HbA1c, self-management misunderstanding, FPG, 2-h PBG, QoL	PRECEDE-PROCEED model is effective in correcting self-management misconceptions of elderly diabetics, lowering blood glucose levels and improving quality of life
Li 2018 China [25]	RCT	152	153	IG: 65.1 ± 13.6 CG: 64.3 ± 12.0	6 months three telephone follow-ups (every 2 months)	Endocrinology department	Health knowledge lectures including PPT presentations, case sharing, group discussions and online media to reinforce self-management behaviors, and follow-up to consolidate effects	End of the 6-months follow-ups	HbA1c, self-management, lipids levels, BP	PRECEDE-PROCEED model-based health education can enhance the self-management behaviour and reduce HbAlc, blood pressure of patients with T2DM
Hu et al. 2015 China [12]	RCT	103	103	IG: 52.0 C 4.3 CG: 53.7 ± 4.1	not reported 2 h-group lectures twice a month	Community	Community strengthening management including group lectures mainly in the form of lectures, videos or VCDs, personalised health education; family doctor visits and counselling	End of the intervention	HbA1c, lipids levels, BP	PRECEDE-PROCEED mode-based community reinforcement management can effectively reduce final systolic blood pressure, FBG, 2-h PBG, HbA1c, and LDL levels in patients with T2DM
Barasheh et al 2017 Iran [26]	QE	55	55	IG: 48.74 ± 8.81 CG: 49.89 ± 7.9	3 months four 60- minute educational sessions (twice a week)	Health centers	Lectures with question-and-answer sessions, group discussions, practical presentations, educational films and printed materials	1 months post-intervention	Self-management, PP model variables, BMI	Precede-Proceed model was a suitable conceptual structure to enhance PP model variables in patients with T2DM.
Ebadifard et al 2017 Iran [10]	QE	43	43	IG: 56.65 ± 10.76 CG: 55.09 ± 13.41	2 months eight 45-to-60 min educational sessions	Diabetic clinics	Based on literature review and focus groups, implement educational sessions about healthy eating, exercise and medication	1 and 3 months post-intervention	PP model variables, QoL	PRECEDE-PROCEED model-based education improved PP variables, quality of life and self-management in type 2 diabetics
Ebadifard et al 2018 Iran [9]	QE	43	43	IG: 55.09 ± 13.41 CG: 59.09 ± 15.86	not reported eight 60- to 90-min educational sessions	Diabetes clinics	Integrating self-management education with the PRECEDE-PROCEED model—addressing areas like diet, physical activity, medication adherence, blood glucose monitoring and foot care	1 month post-intervention	PP model variables	PRECEDE-PROCEED model-based education, whether used alone or alongside self-management theory, can enhance predisposing, enabling, and reinforcing factors inpatients with T2DM

*CG* Control group, *FPG* Fasting blood glucose, *HbA1c* glycated hemoglobin, *IG* Intervention group, *PP* model variables: knowledge, attitudes, self-efficacy, reinforcing factors, and enabling factors, *QoL* Quality of life, *RCT* Randomized Controlled Trial, *QE* Quasi-experimental study, *2-h PBG* 2-h postprandial blood glucos

### Quality assessment of included studies

**Fig. 2:**
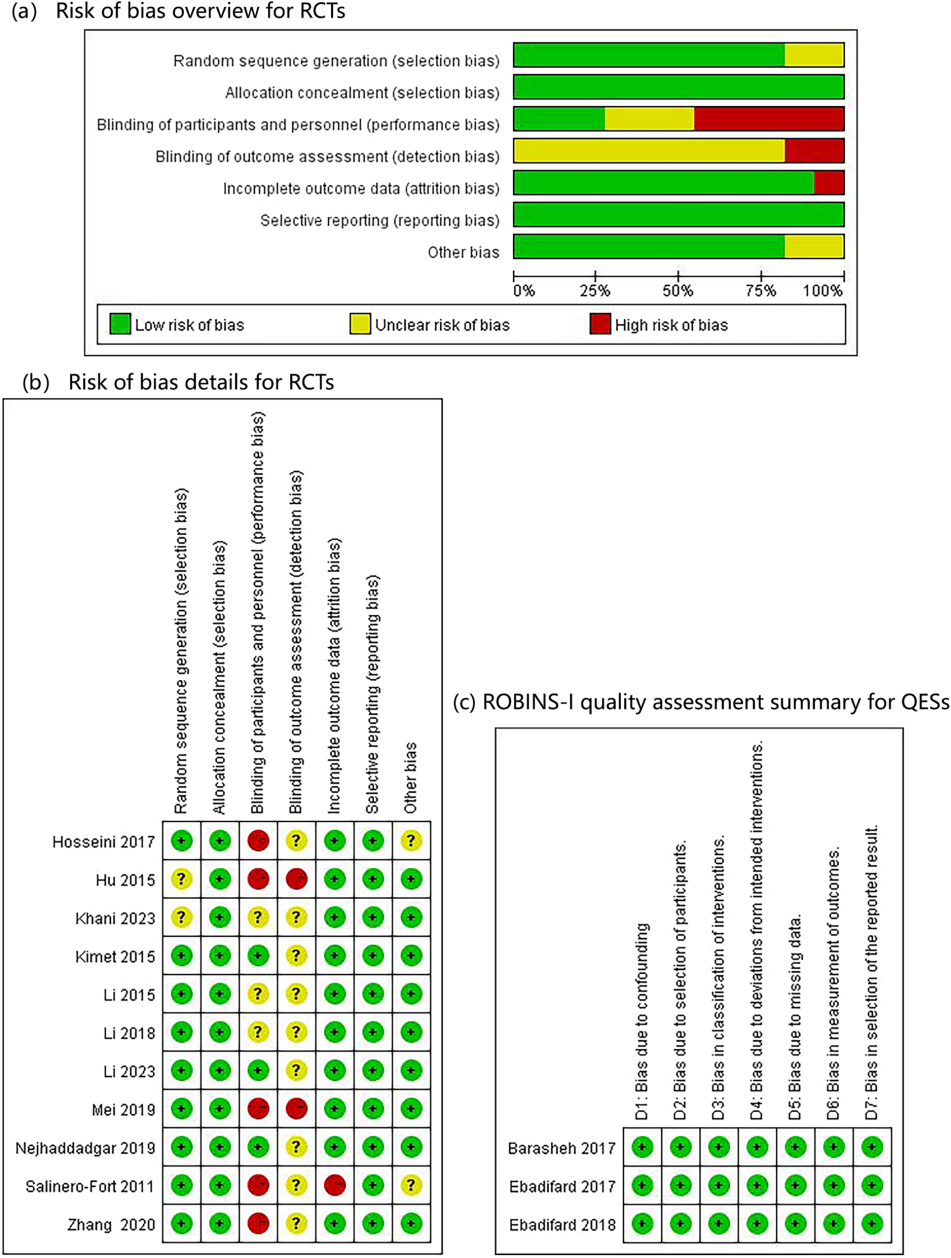
Quality assessment assessed and risk bias

As systematically evaluated in Fig. 2, the 11 RCTs demonstrated: (1) strong methodology in random sequence generation (81.8% low risk, with 9/11 studies using computer-based randomization) and allocation concealment (100% low risk, using sealed envelopes), but unavoidable limitations in blinding-related domains—performance bias was rated high or unclear in 72.7% (8/11) due to the constraints of behavioral interventions, and detection bias was rated as unclear in 100% of the studies due to insufficient documentation of assessor blinding. Other domains, including incomplete outcome data (< 15% attrition) and selective reporting, consistently showed low risk. The 3 quasi-experimental studies (assessed with ROBINS-I) achieved uniformly low risk across all domains, particularly excelling in confounding control (matched cohorts), participant selection (prespecified criteria), and outcome measurement (standardized protocols), demonstrating a rigorous non-randomized study design.

### Description of interventions

In 13 studies, interventions for the experimental groups primarily comprised of educational programs based on the PRECEDE-PROCEED framework, while control groups received standard educational content. One study [9] uniquely combined self-management theory with the PRECEDE-PROCEED framework for the experimental group, using the PRECEDE-PROCEED model alone for the control group. Various instructional methods were utilized, including lectures, group discussions, demonstrations, peer and family support, and distribution of educational materials. The duration of interventions ranged from one month [21] to two years [22], with individual sessions lasting between 40 and 120 min. Session frequencies varied, including weekly [11, 18, 21], biweekly [10, 20, 26], and monthly [19] schedules and follow-up sessions every two months [25] or three months [22]. Healthcare professionals, such as nurses, doctors, and community health educators, delivered most interventions.

### Narrative synthesis of outcomes

The primary outcomes were HbA1c levels and self-management, while secondary outcomes included key PRECEDE-PROCEED model variables (knowledge, attitudes, self-efficacy, reinforcing factors, and enabling factors), quality of life, and physiological indicators. The included studies demonstrated consistent improvements in primary outcomes. Nine studies [17–19, 21–26] reported statistically significant reductions in HbA1c levels across varying follow-up periods (from immediate post-intervention to two-year follow-up). Self-management capacities showed robust enhancement in eight studies, though measurement tools varied, which could account for some differences in the reported effects: three studies [18, 23, 24] used the validated SDSCA scale [27], two [11, 25] employed study-specific instruments, and three studies utilized established questionnaires: Chinese CDC questionnaire [19], Walker and Pender's instrument [20], Glasgow Diabetes Self-care questionnaire [12]. Notably, HbA1c improvements correlated with self-management gains in five studies [18, 19, 23, 24, 26], suggesting behavioral mechanisms may underlie glycemic control benefits.

Intervention effects extended to PRECEDE-PROCEED model variables and clinical biomarkers. Five studies [9–12, 20] confirmed significant improvements in knowledge, attitudes, self-efficacy, reinforcing and enabling factors—key PRECEDE-PROCEED model variables. Physiologically, four studies [17, 19, 23, 24] documented concurrent reductions in 2 h-PBG and FPG, with three showing HbA1c synergy. Metabolic outcomes exhibited variability, with only 1/5 BMI studies [21] and 2/4 lipid studies [25, 26] reaching statistical significance, while 3/6 blood pressure studies [20, 25, 26] demonstrated clinically meaningful reductions. This pattern suggests the intervention most consistently affected glucose-related parameters, with variable impacts on other cardiometabolic risk factors.

Differences in study design, such as PRECEDE-PROCEED phase application and measurement tools, may explain the outcome discrepancies. Thirteen studies fully applied all PRECEDE-PROCEED phases (planning/implementation/evaluation), while one [19] focused solely on planning. Measurement tool heterogeneity was evident, with six studies using model-specific scales (one standardized [28], five custom-developed) and eight incorporating supplementary measures (e.g., quality-of-life scales). Crucially, studies employing standardized tools (e.g., SDSCA, Dizaji's scale) showed more consistent effects, suggesting measurement precision influences outcome interpretation. The 6-month assessment window (used in 6/14 studies) appeared optimal for detecting sustained effects, especially when compared to the longer follow-up periods observed in some studies.

### Results of meta-analysis

#### Effect on HbA1c

**Fig. 3:**
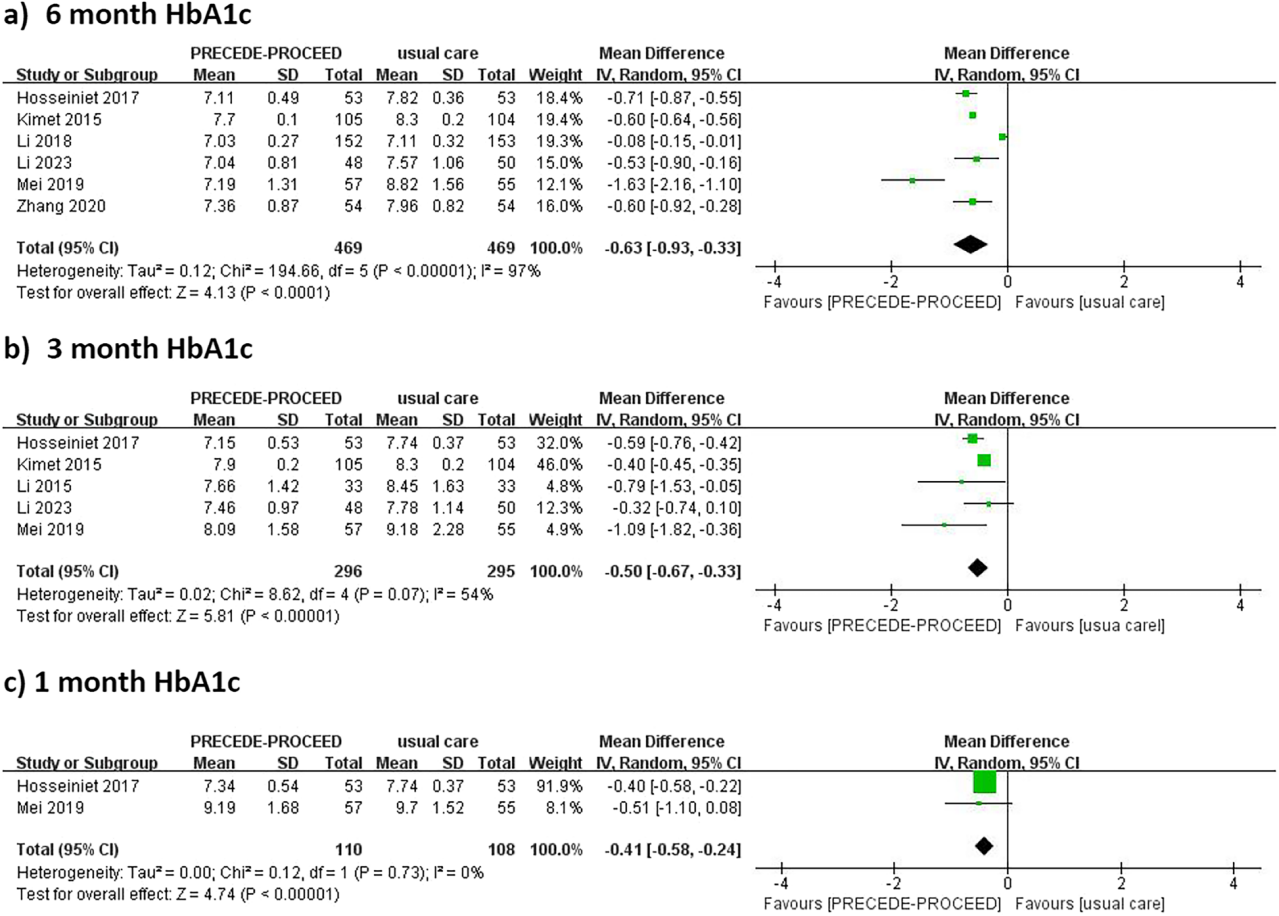
Forest plot of HbA1c

Six studies reported HbA1c outcomes at 6-month follow-up [18, 19, 21, 23–25]. The intervention group showed significantly lower HbA1c at 6 months (WMD = −0.63; 95% CI: −0.93 to −0.33; *p* < 0.0001) with substantial heterogeneity (*I*^2^ = 97%). To explore sources of heterogeneity, sensitivity analysis identified Li [25] as the primary contributor—its exclusion reduced *I*^2^ to 75% (WMD = −0.71; 95% CI: −0.95 to −0.47). This can be explained by the distinctly lower baseline HbA1c (intervention: 7.03 ± 0.27% vs. the range of 7.04–8.82% in other studies), while exclusions of other studies maintained high heterogeneity (*I*^2^ 95–98%) (complete sensitivity analysis data available upon request). For 3-month outcomes, the analysis (*n* = 5 studies) demonstrated a significant reduction (WMD = −0.50; 95% CI: −0.67 to −0.33; *p* < 0.00001) with moderate heterogeneity (*I*^2^ = 54%). Exclusion of Kim [18] (SDs: intervention 0.1/control 0.2, indicating the highest measurement precision) or Hosseini (2017) [21] (SDs: intervention 0.49/control 0.36) reduced *I*^2^ to 19% and 35%, respectively, suggesting that measurement variability is a key factor, while exclusions of other studies showed consistent moderate heterogeneity (*I*^2^ 44–64%). Finally, the 1-month subgroup (*n* = 2) confirmed intervention benefits with perfect consistency (WMD = −0.41; 95% CI: −0.58 to −0.24; *I*^2^ = 0%), demonstrating early effects prior to the observed heterogeneity at later time points (Fig. 3).

#### Effect on self-management

**Fig. 4:**
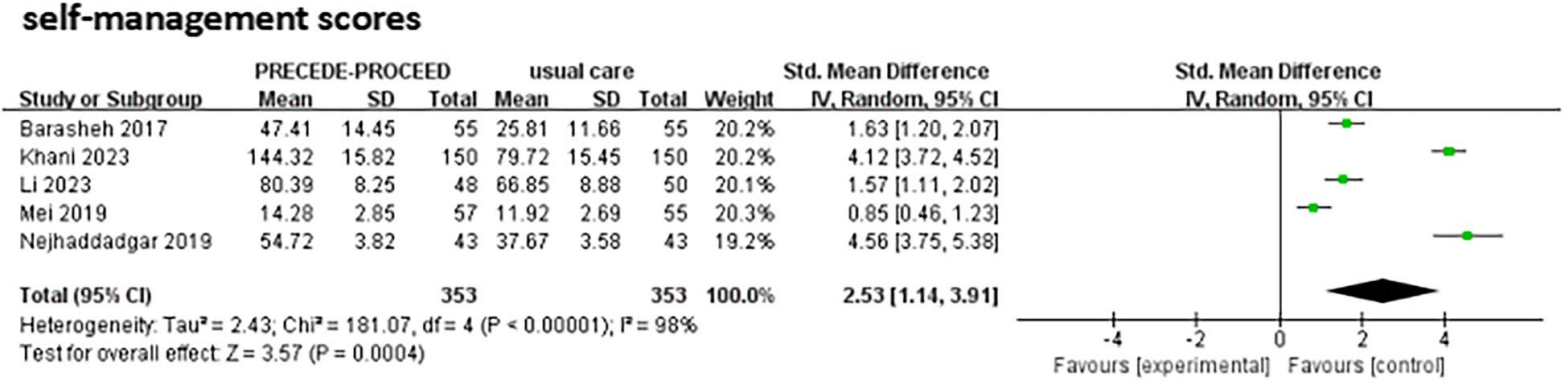
Forest plot of total self-management scores

Eight studies [11, 12, 18–20, 23–25] reported significant improvements in self-management domains, including diet, exercise, blood glucose monitoring, and foot care. Due to variations in evaluation scales and interpretations across studies, five studies reporting total self-management scores were included in meta-analysis [11, 12, 19, 20, 23]. The standardized mean difference (SMD) was used to account for these methodological differences. The meta-analysis revealed a statistically significant improvement in total self-management scores for the intervention group compared to the control group (SMD = 2.53; 95% CI: 1.14 to 3.91; *p* = 0.0004),, with substantial heterogeneity (*I*^2^ = 98%; Fig. 4). Sensitivity analysis by sequentially excluding individual studies confirmed persistently high heterogeneity (*I*^2^ > 95%), likely attributable to the variability in evaluation scales. The wide range of effect sizes (individual study estimates: 0.85 to 4.56) further supports the intervention's impact, albeit with inconsistent magnitude across studies.

#### Effect on PRECEDE-PROCEED model variables

**Fig. 5:**
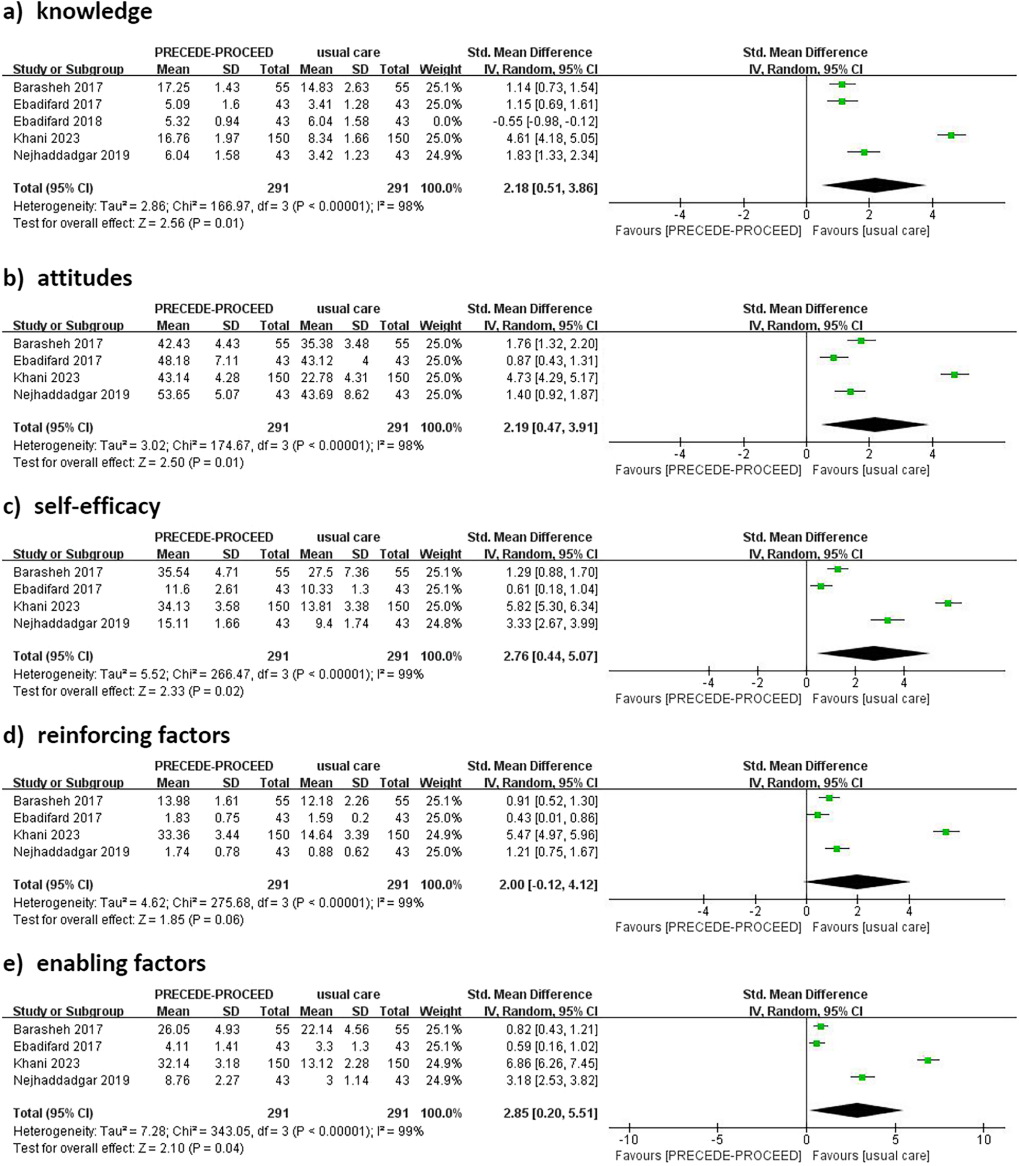
Forest plots of PRECEDE-PROCEED model variables

Data on the five key PRECEDE-PROCEED model variables—knowledge, attitudes, self-efficacy, reinforcing factors, and enabling factors—were reported in six studies. Among these, one study [21] presented outcomes as percentages, while another [9] integrated the model with self-management theory and compared it to a control group using only the PRECEDE-PROCEED model. Therefore, four studies [10–12, 20] using researcher-developed questionnaires were included in the meta-analysis, with SMD employed as the effect size. Endpoint values were used to enhance statistical power. The meta-analysis demonstrated substantial improvements in all five variables for the intervention group compared to the control group: knowledge (SMD = 2.18; 95% CI: 0.51 to 3.84; *p* = 0.01; *I*^2^ = 98%), attitudes (SMD = 2.19; 95% CI: 0.47 to 3.91; *p* = 0.01; *I*^2^ = 98%), self-efficacy (SMD = 2.76; 95% CI: 0.44 to 5.07; *p* = 0.02; *I*^2^ = 99%), reinforcing factors (SMD = 2.00; 95% CI: 0.12 to 4.12; *p* = 0.05; *I*^2^ = 99%), enabling factors (SMD = 2.85; 95% CI: 0.20 to 5.51; *p* = 0.04; *I*^2^ = 99%).These results are illustrated in Fig. 5.

After excluding the study [20] with the largest sample size, heterogeneity for knowledge, attitude, and reinforcing factors significantly decreased, with *I*^2^ values dropping to 62%, 74%, and 67%, respectively, all below the 75% threshold. The overall effect sizes for these variables remained statistically significant, indicating that this study contributed substantially to the observed heterogeneity. However, heterogeneity for self-efficacy and enabling factors showed minimal change, suggesting that the variability in these domains may stem from differences in study design.

## Discussion

This meta-analysis systematically evaluated the effects of PRECEDE-PROCEED model-based interventions on HbA1c levels and self-management among patients with T2DM. The analysis included pooled data from 14 studies conducted across four countries involving a total of 2478 participants. The findings demonstrated that the PRECEDE-PROCEED model significantly improves both HbA1c levels and self-management behaviors, emphasizing its potential as a practical framework for diabetes education and management.

The included studies were conducted across diabetes clinics (*n* = 5), endocrinology departments (*n* = 4), and communities (*n* = 3), with differential effects observed. Clinic-based interventions resulted in larger HbA1c reductions (WMD = −0.72) compared to community programs (WMD = −0.39), likely due to stricter protocol adherence and immediate clinical feedback. Additionally, cultural context played a role in outcomes, as Iranian studies reported higher self-efficacy gains (SMD = 3.12) than studies from China (SMD = 1.89). This difference may reflect the collectivist cultural reinforcement of peer support [12, 18], aligning with Doshmangir's (2023) research [29] on the importance of context-dependent model adaptation. The PRECEDE-PROCEED model, as proposed by Lawrence W. Green, offers a structured framework that includes multi-level assessment, systematic intervention implementation, and guided evaluation. This model supports health behavior change and informs health promotion strategies in diverse populations and contexts [7]. The model's application was organized into three distinct phases: planning, implementation, and evaluation, with educational diagnosis serving as its core component [7, 19]. By identifying and addressing predisposing, reinforcing, and enabling factors, the model demonstrates its capacity to facilitate and sustain behavioral change [7, 30].

Predisposing factors identified in the studies included knowledge, attitudes, complications, and methods for managing specific diseases. Enabling factors encompassed access to resources and skills, such as professional guidance, available services and resources [7]. Reinforcing factors involved support from family or friends [31] and tailored expert management [12]. Despite variability in assessment methods, 13 studies shared a consistent explanation of these three factors. However, one study [18] offered a different perspective, proposing that predisposing factors also include demographic information, lifestyle-related behaviors, diabetes risk factors, medical history, and depression. Study [18] defined enabling factors as diabetes knowledge and self-efficacy, while reinforcing factors were not mentioned. Hansen et al. [32] recently underscored the importance of understanding the concepts and constructs of theoretical models to ensure their effective application. Accordingly, research grounded in theory or models should prioritize providing comprehensive and high-quality descriptions of educational intervention frameworks. Future research should also aim to develop standardized methods for designing assessment tools and defining variables. Additionally, exploring how these factors may shape the model's effectiveness across different cultural contexts could provide valuable insights.

All studies included in this meta-analysis involved educational interventions targeting specific populations and providing practical recommendations for health program administrators [7, 33]. Most studies employed multiple approaches, with group-based teaching and regular follow-ups being the most common. Group-based education addressed predisposing factors through diverse formats, such as health lectures, workshops, discussions, and interactive Q&A sessions. These formats facilitated peer learning and interaction [34], enhanced participants'knowledge, encouraged experience sharing, and fostered positive attitudes towards disease management [35]. Supportive interventions—such as regular home visits (in person or via telephone), peer support groups, and outpatient professional counselling—provided essential social support, maintaining participants'engagement and motivation in health management. Additionally, tools such as educational materials, monitoring diaries, and practical guidance on self-monitoring blood glucose, physical exercise, and dietary adherence addressed enabling factors by equipping participants with essential self-management skills [36]. These findings underscore the importance of a comprehensive approach, demonstrating that multifaceted interventions can drive sustained behavioral changes and improved health outcomes in individuals with T2DM, consistent with previous research [34].

This study supports previous systematic reviews [29, 34, 37], demonstrating the effectiveness of PRECEDE-PROCEED model-based interventions in reducing HbA1c levels in type 2 diabetes patients. It also offers new insights into the model's role in enhancing self-management behaviors and improving its key variables. The meta-analysis revealed significant HbA1c reductions at 1, 3, and 6 months, with effect sizes of −0.41, −0.50, and −0.63, respectively, indicating sustained benefits and progressive improvements over time. Heterogeneity was notably high at the 6-month follow-up period (*I*^2^ = 97%), which may be attributed to two key methodological variations across studies. First, population diversity in baseline characteristics (e.g., age range 48.7–68.1 years) likely influenced intervention responsiveness. Second, differences in intervention protocols—including session frequency (weekly to monthly), total duration (1 month to 2 years), and implementers (nurses vs. multidisciplinary teams)—may have contributed to effect size disparities. Shorter follow-up intervals exhibited lower heterogeneity (1 month: *I*^2^ = 0%, 3 months: *I*^2^ = 54%), suggesting that individual characteristics and adherence might vary over time. Previous studies [38, 39] have shown that lower HbA1c levels are associated with a reduced risk of microvascular and macrovascular complications, emphasizing the PRECEDE-PROCEED model's potential as an effective framework for glycemic control and complication prevention, making it a promising approach for T2DM management.

Pooled analysis showed substantial improvements in self-management (SMD = 2.53, 95%CI: 1.14–3.91), though with extreme heterogeneity (*I*^2^ = 98%) primarily due to incompatibilities in measurement tools (e.g., SDSCA vs. study-specific questionnaires). Additional variability stemmed from differing behavioral constructs (comprehensive behaviors vs. diet-focused interventions) and varying assessment timings (immediate post-intervention vs. 3–6 month follow-ups), highlighting the urgent need for standardized diabetes self-management metrics in future trials. Despite these methodological variations, all studies reported positive effects, emphasizing the intervention's robust impact on behavior.

The PRECEDE-PROCEED model identifies predisposing factors—comprising knowledge, attitudes, and self-efficacy—along with reinforcing and enabling factors as key elements in behavior change [11, 12, 22]. Systematic diagnosis within the model ensures their effective application. This meta-analysis demonstrated significant improvements in the model's five key variables, with effect sizes of 2.18 for knowledge, 2.19 for attitudes, 2.76 for self-efficacy, 2.00 for reinforcing factors, and 2.85 for enabling factors. Among these variables, self-efficacy showed the strongest association with self-management, as noted by Peyrot and Rubin [40]. This finding aligns with the substantial increase in self-efficacy observed in this study, highlighting the intervention's effectiveness in boosting participants'confidence in managing their health, as shown in earlier systematic reviews [41]. However, the high heterogeneity observed (*I*^2^ = 98% to 99%) likely reflects variations in sample sizes and research designs. Sensitivity analyses revealed that excluding the largest study significantly reduced heterogeneity for knowledge, attitude, and reinforcing factors, emphasizing the importance of methodological consistency in future research.

Despite the significant findings, this meta-analysis has certain limitations. First, the heterogeneity of the sample, coupled with variations in intervention duration, frequency, and content across the included studies, complicates direct comparisons. Second, the inability to implement blinding in several studies may have introduced bias, potentially affecting the reliability and generalizability of the results. Additionally, the reliance on self-reported measures for assessing self-management behaviors introduces possibility of reporting bias, which may undermine the credibility of the outcomes. These limitations underscore the need to standardize intervention protocols and establish consistent, objective outcome measures to enhance the reliability and validity of future research.

## Conclusion

This meta-analysis demonstrated that the PRECEDE-PROCEED model-based interventions significantly reduced HbA1c levels and enhanced self-management among patients with T2DM, underscoring the crucial role of structured, theory-driven education in health promotion. Future studies should prioritize the development of high-quality assessment tools and robust study designs to explore the model's applicability across diverse populations and health conditions, thereby reinforcing the effectiveness and sustainability of chronic disease management strategies.

## Data Availability

The datasets analyzed during this systematic review are from previously published studies listed in the references. The extracted and analyzed datasets during the current study are available from the corresponding author on reasonable request.

## Abbreviations

2-h PBG: 2-Hour postprandial blood glucose
FPG: Fasting blood glucose
HbA1c: Glycated hemoglobin
QES: Quasi-experimental study
RCT: Randomized Controlled Trial
T2DM: Type 2 diabetes mellitus
WHO: World Health Organization
